# Predictive value of miRNA-126 on in-stent restenosis in patients with coronary heart disease: A protocol for meta-analysis and bioinformatics analysis

**DOI:** 10.1097/MD.0000000000025887

**Published:** 2021-06-04

**Authors:** Xianke Qiu, Jun Wang, Zhongping Shi, Xiaojun Ji, Yiwei Huang, Haiyue Dai

**Affiliations:** a Department of Emergency Medicine; b Department of Cardiology, Wenzhou Central Hospital, Wenzhou, Zhejiang Province, China.

**Keywords:** bioinformatics, coronary heart disease, in-stent restenosis, meta-analysis, microRNA-126, percutaneous coronary intervention, protocol

## Abstract

**Background:**

In-stent restenosis (ISR) is one of the most important complications and impacts the long-term effects after percutaneous coronary intervention (PCI) in patients with coronary heart disease (CHD). Related studies have revealed that microRNA (miRNA) can predict ISR in CHD patients. MiRNA-126 may be a potential biomarker for the diagnosis of ISR. However, the accuracy of miRNA-126 in the diagnosis of ISR is still controversial. Therefore, this study carried out meta-analysis to further evaluate the accuracy of miRNA-126 in the diagnosis of ISR. At the same time, bioinformatics is used to predict the target genes and miRNA-126 may be involved in regulation, so as to provide theoretical support for the precise treatment of CHD.

**Methods:**

The literatures on the miRNA-126 diagnosis of ISR in CHD patients were collected by searching on computer through China National Knowledge Infrastructure, Wanfang, China Biology Medicine disc, PubMed, EMBASE, Cochrane Library and Web of Science. The retrieval time is set to build the database until April 2021. The meta-analysis of the literatures that meet the quality standards was conducted by Stata 16.0 software. TargetScan database, PicTar database, miRanda database, and miRDB database were used to predict miRNA-126 intersection target genes. Gene Ontology (GO) functional enrichment analysis and Kyoto Encyclopedia of Genes and Genomes (KEGG) signal pathway enrichment analysis of miRNA-126 target genes were performed by using DAVID database. STRING database was applied to analyze the protein-protein interaction (PPI) network of miRNA-126 target genes. The “Networkanalyzer” function of Cytoscape3.7.2 software is adopted to analyze the network topology attributes, so as to find out the core genes of PPI network.

**Results:**

The results of this meta-analysis will be submitted to a peer-reviewed journal for publication.

**Conclusion:**

In this study, meta-analysis and bioinformatics analysis were adopted to further evaluate the accuracy of miRNA-126 in the diagnosis of ISR in CHD patients, and to explore the mechanism of the action of miRNA-126 and understand related pathways.

**Ethics and dissemination:**

The private information from individuals will not be published. This systematic review also should not damage participants’ rights. Ethical approval is not available. The results may be published in a peer-reviewed journal or disseminated in relevant conferences.

**OSF REGISTRATION NUMBER:**

DOI 10.17605/OSF.IO/9FMR5.

## 1 Introduction

Coronary heart disease (CHD) is a kind of cardiovascular disease that is caused by coronary artery stenosis due to coronary atherosclerosis, thus leading to insufficient blood supply of heart and myocardium.^[1–5]^ It is one of the major serious diseases of human death in the world. Percutaneous coronary intervention (PCI) has become 1 of the important methods for the treatment of CHD.^[6,7]^ However, the subsequent occurrence of in-stent restenosis (ISR) seriously affects the efficacy and health of PCI in patients with CHD.^[8,9]^ Although drug-eluting stents can reduce the incidence of ISR in a short term, in the end, the incidence of ISR is still very high,^[10]^ which brings great difficulty to clinical work. For a long time, early prevention, early diagnosis and early intervention treatment of CHD are important links to reduce the morbidity and mortality of CHD.

MicroRNA (miRNA) is a kind of non-coding endogenous small molecule RNA, and it is composed of highly conserved 1825 nucleotides. MiRNA plays an important regulatory role in the physiological and pathological process of human body,^[11]^ and it is involved in human cell differentiation, proliferation, apoptosis, cell metabolism and the occurrence and development of disease.^[12]^ It is known that miRNA is involved in the regulation of a variety of heart diseases, including cardiovascular system development, atherosclerosis, hypertension, arrhythmia, heart failure, cardiovascular interventional therapy for restenosis and so on.^[13–16]^

MiRNA-126 is a short-stranded non-coding RNA and located in epidermal growth factor-like domain 7 gene. It is the most widely studied and the only known class of miRNAs, and it is specifically expressed in endothelial cells and hematopoietic stem cells to play a protective role in vascular endothelium. More and more studies have proved that there is a correlation between miR-126 and the occurrence and development of cardiovascular disease.^[17–19]^ Therefore, miRNA-21 may be a potential auxiliary biomarker for the diagnosis and prognosis of cardiovascular diseases.

Some studies have confirmed that miRNA-126 can predict the occurrence of ISR after PCI, but others concluded negative results.^[20–22]^ In this study, in order to explore whether miRNA-126 can be used as a good biological marker to predict ISR after PCI, meta-analysis was carried out to analyze the relevant research literature at home and abroad. In addition, the target genes of miRNA-126 were predicted by bioinformatics methods, and their target genes were analyzed by gene ontology (GO) enrichment analysis, kyoto encyclopedia of genes and genomes (KEGG) signal pathway enrichment analysis and protein-protein interaction (PPI) network analysis, so as to provide theoretical basis and data support for further researches on ISR.

## 2 Methods

### 2.1 Study registration

The protocol of the systematic review has been registered on Open Science Framework (registration number: DOI 10.17605/OSF.IO/9FMR5). It was reported by following the guideline of Preferred Reporting Items for Systematic Reviews and Meta-analysis Protocol Statement.^[23]^

### 2.2 Inclusion criteria for study selection

#### 2.2.1 Inclusion criteria

1. Type of studies: To explore the diagnostic value of miRNA-126 on the diagnosis of ISR in patients with CHD.
2. Participants: All patients with CHD after PCI were included.
3. Index test: MiRNA-126 was applied to detect patients with ISR.

#### 2.2.2 Exclusion criteria

Case reports, reviews, cell, or animal studies.

### 2.3 Data sources and search strategy

This study conducted a literature search in China National Knowledge Infrastructure, Wanfang, China Biology Medicine disc, PubMed, EMBASE, Cochrane Library, and Web of Science. The retrieval time is set to build the database until April 2021. The language restrictions are Chinese and English. The search strategy for PubMed is displayed in Table 1.

**Table 1:** PubMed search strategy.

Number	Search terms
#1	Coronary Disease[MeSH]
#2	Coronary Heart Disease[Title/Abstract]
#3	Coronary Diseases[Title/Abstract]
#4	Coronary Heart Diseases[Title/Abstract]
#5	Disease, Coronary[Title/Abstract]
#6	Disease, Coronary Heart[Title/Abstract]
#7	Diseases, Coronary[Title/Abstract]
#8	Diseases, Coronary Heart[Title/Abstract]
#9	Heart Disease, Coronary[Title/Abstract]
#10	Heart Diseases, Coronary[Title/Abstract]
#11	or/1–10
#12	Restenosisin-stent[Title/Abstract]
#13	In-stent restenosis[Title/Abstract]
#14	ISR[Title/Abstract]
#15	Coronary Restenosis[MeSH]
#16	Coronary Restenoses[Title/Abstract]
#17	Restenoses, Coronary[Title/Abstract]
#18	Restenosis, Coronary[Title/Abstract]
#19	or/12–18
#20	miRNA-126[Title/Abstract]
#21	microRNA-126[Title/Abstract]
#22	miR-126[Title/Abstract]
#23	or/20–22
#24	diagnos∗[Title/Abstract]
#25	sensitivity[Title/Abstract]
#26	specificity[Title/Abstract]
#27	ROC curve[Title/Abstract]
#28	or/24–27
#29	#11 and #19 and #23 and #28

### 2.4 Data collection and analysis

#### 2.4.1 Study selection

**Figure 1:**
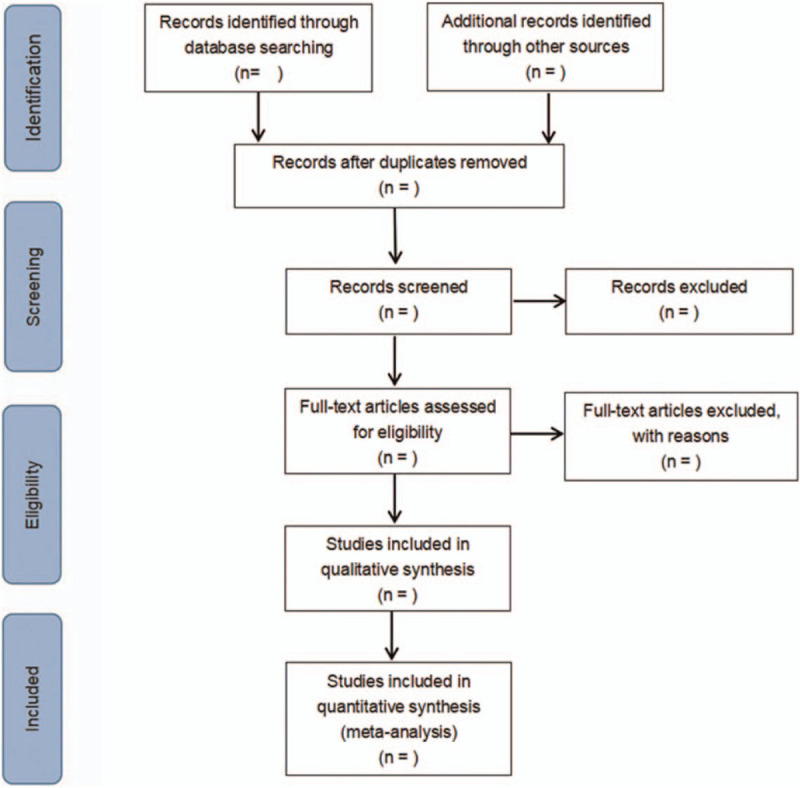
Flow diagram of literature retrieval.

The screening flow chart of this study is demonstrated in Figure 1. The literature was screened independently by 2 researchers based on the inclusion and exclusion criteria. In case of disagreement, a decision is made through discussion or consultation with relevant experts.

#### 2.4.2 Data extraction

The data extraction form includes following items: first author, publication year, regions, sample size, sample types, control group, miRNA-126 detection methods, and data needed for diagnostic meta-analysis.

#### 2.4.3 Dealing with missing data

If there are insufficient or missing data in the literature, the authors will be contacted via email. If the data are still not available, only the current available data will be analyzed and the potential impacts will be discussed.

### 2.5 Quality assessment

The methodological quality of the included studies was assessed by following Quality Assessment of Diagnostic Accuracy Studies-2 criteria.

### 2.6 Statistical analysis

All of the above statistical analyses were performed with Stata 16.0 (Stata Corp LLC, college station, TX, USA). We calculated the pooled sensitivity (SEN), specificity (SPE), positive likelihood ratio, negative likelihood ratio, diagnostic odds ratio (DOR), area under the curve, and their 95% CI. In addition, the pooled diagnostic value of miRNA-126 through the summary receiver operating characteristic curve (SROC) and area under the curve was tested. The threshold effects were detected by applying spearman correlation coefficient. The calculation of heterogeneity was caused by the non-threshold effect of Cochrane-Q and *I*^2^ values, and a fixed effect model (without obvious in homogeneity) or a random effects model (with significant heterogeneity) was employed to merge the data. The statistical test level was α=0.05.

### 2.7 Subgroup analysis

A subgroup analysis will be made on the basis of ethnicity, the source of miRNA-126 and sample size.

### 2.8 Sensitivity analysis

We will adopt the one-by-one exclusion method to analyze the sensitivity of the results.

### 2.9 Reporting bias

The publication bias was determined by conducting Deeks’ funnel plot asymmetry test.

### 2.10 Ethics and dissemination

Since the program does not include the recruitment of patients and the collection of personal information, it does not require the approval of the Ethics Committee.

### 2.11 Bioinformatics analysis

#### 2.11.1 MiRNA-126 target gene prediction

The target genes of miRNA-126 were predicted by TargetScan database (http://www.targetscan.org/mamm_31/), PicTar database (https://pictar.mdc-berlin.de/), miRanda database (http://www.miranda.org/), and miRDB database (http://mirdb.org/). The intersection of each database was taken and the Wayne diagram was made to obtain the common target genes.

#### 2.11.2 GO functional annotation and KEGG enrichment analysis of miRNA-126 target gene

The DAVID database (https://david.ncifcrf.gov/) was used to annotate the GO function of the target gene of miRNA-126, and its contents include the biological process of gene participation, molecular function and cell composition. At the same time, the KEGG pathway enrichment analysis of target genes was carried out to find out the signal transduction pathways with significant differences. The difference was statistically significant (*P* < .05).

#### 2.11.3 Construction of protein-protein interaction (PPI) network

The intersection target of miRNA-126 was imported into STRING software (https://string-db.org) to obtain the protein-protein interaction network. Saving the TSV data file, importing the “Networkanalyzer” function of Cytoscape3.7.2 software to analyze the network topology attributes, and finding out the core genes of PPI network.

## 3 Discussion

At present, the most reliable method for vascular stenosis caused by CHD is PCI on the basis of drug treatment. On the contrary, after operation, there is often recurrence of the disease, and the emergence of ISR. It is generally believed that ISR is an injury and a kind of repair response of blood vessels around the stent, including intimal hyperplasia, negative remodeling and thrombosis.^[24,25]^ At present, more and more studies have confirmed that miRNA plays an important role in vascular restenosis.^[26–28]^

The specific expression of miRNA-126 in vascular endothelial cells has been confirmed. The down-regulation of its expression can increase the activity of cell adhesion molecule-1, promote the formation of neovascularization and maintain the integrity of blood vessel wall.^[29]^ Most importantly, miRNA-126 regulates the expression of vascular adhesion cytokine-1, thus affecting the development of atherosclerotic plaque.^[30]^

Many studies have proved that the expression of miRNA-126 is abnormal in patients with ISR after PCI. We conducted a meta-analysis to determine the accuracy of miRNA-126 in the diagnosis of ISR in patients with CHD to resolve disputes. On the basis of meta-analysis, bioinformatics analysis methods were further adopted to explore the potential mechanism of miRNA-126, so as to provide reference for follow-up molecular biology research.

## Author contributions

**Conceptualization:** Haiyue Dai, Xianke Qiu.

**Data collection:** Jun Wang, Zhongping Shi.

**Data curation:** Xianke Qiu, Jun Wang.

**Funding acquisition:** Haiyue Dai.

**Funding support**: Haiyue Dai.

**Methodology:** Yiwei Huang.

**Project administration:** Haiyue Dai.

**Resources:** Haiyue Dai.

**Software:** Jun Wang, Xiaojun Ji.

**Supervision:** Xiaojun Ji.

**Validation:** Zhongping Shi.

**Visualization:** Zhongping Shi.

**Writing – original draft:** Haiyue Dai, Xianke Qiu.

**Writing – review & editing:** Haiyue Dai, Xianke Qiu.
